# Strengthening health systems for access to gene therapy in rare genetic disorders

**DOI:** 10.1016/j.omtm.2024.101220

**Published:** 2024-03-13

**Authors:** Sonal Bhatia, Yann Le Cam, Juan Carrion, Lauren Diamond, Paul Fennessy, Safiyya Gassman, Felix Gutzwiller, Stephen Kagan, Diana Pankevich, Jennifer Young Maloney, Nitin Mahadev, Martin Schulz, Durhane Wong-Rieger, Paolo Morgese

**Affiliations:** 1 Pfizer, Inc., New York, NY, USA; 2 EURORDIS, Paris, France; 3 Aliber, Totana, Spain; 4 Paul Fennessy Advisory, Melbourne, VIC, Australia; 5 University of Zurich, Zurich, Switzerland; 6 Canadian Organization for Rare Disorders, Toronto, ON, Canada; 7 Alliance for Regenerative Medicine, Brussels, Belgium

## Main text

**Figure 1:**
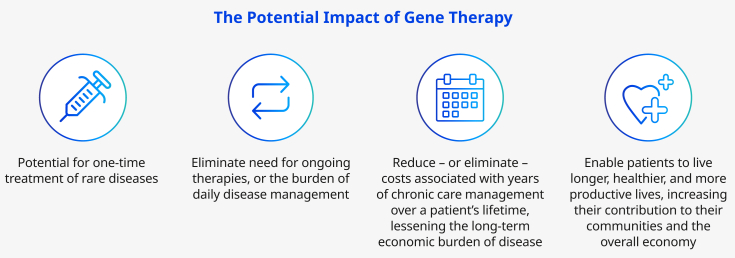
The potential of gene therapy

After decades of innovation, the promise of gene therapy is becoming a reality for patients with certain rare genetic disorders. Several cell and gene therapies have already been approved by regulators worldwide,^1^ with many more expected at a rate of up to 10%–20% annually by 2025.^2^ These therapies have the potential to be transformative, changing the course of a disease and reducing the long-term patient and health system burdens associated with chronic disease management (Figure 1).^2^

Around 80% of rare diseases are genetic in origin,^3^ making them ideal targets for gene therapy. With an estimated 10,000 rare diseases affecting over 300 million people worldwide, they are considered a public health priority in many regions.^3^ While the definition of rare disease varies, they are characterized by low incidence (e.g., 1 in 2,000 in Europe) and often have degenerative and irreversible disease progression.^4^ As such, they place a heavy burden on patients, families, and caregivers, with disease management incurring substantial resource utilization and costs for health systems.^4^

Gene therapies have the potential to transform the lives of people living with devastating rare diseases. However, patient access to these new therapies is not straightforward. In October 2022, Pfizer and the Alliance for Regenerative Medicine convened a roundtable at the World Health Summit in Berlin, Germany, with representatives across stakeholder groups —including academia, civil society, industry, patient communities, healthcare professionals (HCPs), and governments to discuss these topics. Here, we present a summary of barriers and opportunities to accessing gene therapies identified at the roundtable (Table 1), which are described in a report titled “A Framework to Strengthen Health Systems for Gene Therapy for Rare Genetic Diseases.”

**Table 1:** Barriers and opportunities regarding real-world access to gene therapies

Barriers	Opportunities to address barrier
Lack of infrastructure for diagnosis and determining patient eligibility for gene therapy	• Support multistakeholder education • Strengthen infrastructure and workforce for newborn screening (NBS) • Develop governance frameworks supporting eligibility and referrals
Lack of infrastructure for gene therapy delivery	• Certify national clinical centers of excellence (COEs) • Create integrated hub-and-spoke care pathways • Connect COEs across countries
Challenges for the appropriate value assessment for gene therapies and adoption of innovative financing models	• Engage in early dialogue across all stakeholders, including regulators, payers, healthcare professionals (HCPs), biopharma, patient advocacy groups (PAGs), and policymakers • Review value assessment methodologies to ensure processes are fit for gene therapies • Support multistakeholder models of advanced therapy development (e.g., academic, non-profit entities, etc.) • Support the use of novel payment models

## Lack of infrastructure for diagnosis and determining patient eligibility for gene therapy

Early diagnosis and intervention offer the largest potential to delay disease progression for people with rare genetic disorders. However, individuals typically face a complex and lengthy path to diagnosis,^5^ which can be caused by a lack of awareness among patients, families, and HCPs, a lack of trained therapeutic area specialists, and a lack of access to the appropriate diagnostic tests.^5^ For example, in Latin America, the average number of years among those who have suffered a delay in diagnosis of a rare disease is 8.9, with misdiagnoses along the way.^6^ These misdiagnoses can result in additional burden for patients and health systems and delays in care, sometimes with serious or fatal consequences.

A particularly cost-effective method for preventing delays in diagnosis for some genetic conditions is newborn screening (NBS).^7^ Recent studies have shown that inclusion of NBS, coupled with early intervention, is cost effective in comparison to current diagnostic and treatment practices for some diseases.^7^ Greater investment in infrastructure for NBS could reduce the burden on patients and health systems and facilitate timely access to interventions.

Following diagnosis, determining eligibility for gene therapy poses further challenges to access. Assessing eligibility requires evaluation of multiple factors, such as age, disease status, general health status, previous or current therapies, and the presence of neutralizing antibodies.^8^ Given the likely clinically restricted time windows for many gene therapies, development of clear governance frameworks for eligibility referrals and timely diagnostic tests is critical for effective access to gene therapy.

These frameworks need to be multifactorial, encompassing regulatory and reimbursement policies. Recent experience with Zolgensma in Australia saw pediatric hospitals facing financial issues due to existing procurement and reimbursement policies, which resulted in delayed patient access to Zolgensma. As such, government-level horizon scanning of gene and cell therapies in the clinical trial pipeline with support from regulators and payers is needed to drive access processes, ensuring governance frameworks are in place following regulatory approval and reimbursement.

## Lack of infrastructure for gene therapy delivery

The care pathway for gene therapies is complex, requiring a multidisciplinary team and coordination with specialized healthcare providers.^9^ For many rare diseases, clinical centers of excellence (COEs) have been established to facilitate the diagnosis, care, and treatment of patients with ultra-rare conditions.

In addition to centralized, stand-alone COEs, another approach to building these care pathways is the “hub-and-spoke” model, which connects community healthcare practices with specialized clinical COEs or specialized gene therapy treatment facilities. These COEs may be referred to differently across geographies, and there will be a variety of ways in which hubs and spokes work together, though the unifying principles will remain the same: providing patient access to expert and specialist facilities while providing relevant care locally.^10^ For example, COEs could manage the immediate post-infusion time period, with long-term follow-up and surveillance occurring at local practices. Building such hub-and-spoke networks may also allow access to and sharing of resources, such as diagnostic testing, facilitating budgetary efficiencies, and streamlining care delivery. To scale up this innovative approach and facilitate hub-and-spoke implementation, policy frameworks are needed to standardize models of care, ensure the availability of qualified COEs, and ensure reimbursement.

## Challenges with appropriate value assessment and adoption of innovative financing models

The value of a particular gene therapy for reimbursement and access will depend on multiple factors, including safety, efficacy, the durability of clinical benefits, the size of the treatable population, and the financial impact on health systems and patients. Following regulatory approval, evaluation of comparative effectiveness for gene therapies is often complicated by uncertainties due to small clinical trial sizes, lack of appropriate direct comparators, and the need for long-term follow-up.^1^

Traditional health technology assessment (HTA) methodologies cannot capture the full potential value of gene therapies to patients, health systems, and society, as they are not able to appropriately account for long-term impact on healthcare expenditures following one-time administration. As such, the development of methodologies with greater flexibility that can appropriately incorporate estimates of future benefits is required to support reimbursement decisions, for example incorporating a lifetime perspective in modeling and utilizing appropriate discount rates in budget sensitivity.^1^

Beyond methodologies, alignment is needed on assessment criteria and long-term real-world data requirements for gene therapy assessments. Establishment of a formal process for early dialogue among payers, HTA bodies, and manufacturers would facilitate alignment on feasible evidence packages for submission. Post-launch processes for addressing uncertainty and enabling reassessment should also be established, including robust real-world evidence generation and indirect comparisons with other therapeutic options.

Many of the same uncertainties that pose challenges for assessing value appropriately during reimbursement decisions also make it difficult for health systems to pay the upfront prices of some gene therapies while sustaining healthcare budgets. As such, novel payment models that spread payment over time and link to real-world outcomes could help payers to manage the budget impact of gene therapies while recognizing the innovative one-time approach.^1^^,^^11^ Piloting innovative outcomes-based agreements, updating legal and regulatory frameworks, and sharing best practices around innovative financing would be useful to facilitate access to gene therapies.^11^ To enable this, registries need to be identified or established to collect patient outcome data to evaluate the safety and durability of gene therapies over time.

## Conclusion

With several gene therapies now approved by health regulators globally and with more expected to be approved over the coming years,^1^ focus must now be placed on how individuals with rare genetic disorders can achieve timely access to these potentially transformative therapies and what health systems need to due to enable this. Patient centricity is key and is essential to co-create solutions with patients. Collaboration among all stakeholders, including HCPs, patient advocacy groups, policymakers, biopharma, and payers is imperative to ensure that health systems and societies are fully prepared to adopt gene therapies sustainably.
